# Associations between social burden, perceived stress, and diurnal cortisol profiles in older adults: implications for cognitive aging

**DOI:** 10.1007/s10433-021-00616-8

**Published:** 2021-03-28

**Authors:** Anna Pretscher, Saskia Kauzner, Nicolas Rohleder, Linda Becker

**Affiliations:** Department of Psychology, Chair of Health Psychology, Friedrich-Alexander University Erlangen-Nürnberg, Nägelsbachstr. 49a, 91052 Erlangen, Germany

**Keywords:** Social burden, Cognitive aging, Executive functioning, Diurnal cortisol, Stress

## Abstract

Stress can be a risk factor for mental and physical health. We investigated the role of social burden as a potential stressor in older age and its associations with cognitive aging (i.e., within the domains inhibition and cognitive flexibility of executive functioning). Eighty-three older adults (*N* = 38 male, mean age = 74.0 ± 5.7 years, body-mass index = 25.5 ± 4.1 kg/m^2^) aged 64 years or older participated. Cognitive flexibility and inhibition were assessed by means of a Trail-Making Test (TMT) and a Stroop task. Perceived levels of social burden and perceived stress in general were assessed via standardized questionnaires. Furthermore, diurnal cortisol profiles were assessed. Overall, older participants showed lower cognitive performance than the younger (both inhibition and cognitive flexibility; *p* < .044). However, this association did not remain significant when adjusting for multiple comparisons. Social burden was highly correlated with perceived stress, highlighting its importance as a stressor (*p* < .001). Furthermore, participants who reported higher levels of perceived stress showed lower performance in the TMT as a measure for cognitive flexibility (*p* < .001). Moreover, social burden was associated with cognitive flexibility, which was assessed by means of the TMT (*p* = .031). However, this association did not remain significant when using the adjusted α-level. Furthermore, we found that total diurnal cortisol output was a mediator for the relationship between age and inhibition, which was assessed by means of a Stroop task. At last, we found that the mediating role of cortisol interacts with social burden in the way that especially in younger age, high levels of social burden are associated with higher cortisol levels and, therefore, with lower cognitive performance. We conclude that it is important to consider social burden as an additional stressor in older age which is associated with cognitive aging.

**Supplementary Information:**

The online version contains supplementary material available at 10.1007/s10433-021-00616-8.

## Introduction

Impairments in cognitive functioning (e.g., in executive functioning, EF) are one of the most stressful and challenging burdens in older age (Petermann and Roth 2006). Executive functioning is particularly important for older people to maintain an independent and satisfied lifestyle. Executive functions include multiple skills such as cognitive flexibility, inhibition, and working memory (Miyake and Friedman 2012). Even in older people, who do not develop dementia or mild cognitive impairment, sub-clinical cognitive changes can be found in many cases (Harada et al. 2013). There is a distinct age-related decline in EF, particularly in very old age (Wahl and Schilling 2012). This decline is often accompanied by age-related structural changes in the prefrontal cortex (PFC; Brand and Markowitsch 2004; DeCarli et al. 2005; Pfefferbaum et al. 2013), the main brain structure which is involved in EF. One risk factor that is associated with structural and functional changes in the PFC is stress (Arnsten 2009; McEwen et al. 2016; Murmu et al. 2006), and associations between long-term stress exposure and impairments in cognitive functioning have been found (Chrousos 2009; Mika et al. 2012). Therefore, age is associated with the risk of decreasing cognitive functioning, which might be associated with long-term stress exposure (e.g., in EF; Esch, Stefano et al. 2002; Lupien et al. 2009). From a psychological perspective, stress can be explained with respect to the transactional stress model by Lazarus and Folkman 1987. This model focusses on human–environment interactions. According to the transactional stress model, the subjective evaluation of an event (so-called appraisal) determines whether and how subsequent coping takes place (Lazarus and Folkman 1984, 1987). From a biological perspective, a cascade of physiological responses occurs in stressful situations (e.g., when the person detects a threat; Gaab et al. 2005). These include the activation of autonomous, neuro-endocrine, metabolic, and immune responses. One of the most important systems which are involved in the physiological stress response is the hypothalamic–pituitary adrenal (HPA) axis which triggers the release of glucocorticoids (i.e., the stress hormone cortisol in humans) from the adrenal cortex (e.g., Lupien et al. 2009). This HPA axis response is a typical reaction to various stressful stimuli and burdens (Chrousos 2009; Chrousos and Gold 1998; Gunnar and Vazquez 2001). If the person's resources are sufficient to cope with the stressor, the glucocorticoid levels return to baseline and no long-term health consequences occur. However, if the available resources of the individual are exceeded by the frequency and/or intensity of the stressors, physiological and psychological overload can be the result. This can lead to allostatic overload due to non-sufficient adaptation and coping mechanisms (Juster et al. 2010; McEwen 1998), with the HPA axis being one of the most vulnerable physiological systems for allostatic overload development (Zsoldos and Ebmeier 2016).

A widely used and suitable marker to assess HPA-axis functioning is diurnal cortisol activity, which can be assessed by means of diurnal cortisol profiles, which reflect the daily time course of the concentration of free-cycling cortisol and which can be assessed in saliva, urine, or in blood samples (Reinhardt 2007; Young et al. 2004). In healthy humans, typical diurnal cortisol patterns can be found: Immediately after awakening, cortisol levels increase until they reach a maximum 30–45 min afterwards (so-called cortisol awakening response, CAR; Pruessner et al. 1999; Stalder et al. 2016). After this, a decline in cortisol levels throughout the day can be observed with a minimum in the late evening at bedtime (Miller et al. 2007). Flatter diurnal cortisol slopes have been shown to be related with poorer mental and physical health (Adam et al. 2017). However, the associations between age and HPA axis activity and, thus, diurnal cortisol secretion patterns in older adults have still not been fully understood (Heaney et al. 2010; Veldhuis et al. 2013). Subsequently, although it is well known that glucocorticoids are associated with cognitive functioning, it is still unclear to what extent diurnal glucocorticoid secretion patterns are associated with age-related cognitive decline (e.g., Belanoff et al. 2001; Miller and O’callaghan, 2005).

One special potential permanent stressor, which might be particularly important in older age and during the aging process, but which has often been neglected in research on cognitive aging so far, is stress due to negative social relationships. This can in the long term—like other long-lasting stressors—lead to a recurrent and, thus, long-term changes of physiological stress systems such as the HPA axis, the cardiovascular system, and the immune system (Brooks and Dunkel-Schetter 2011). Therefore, negative social relationships can—like long-term stressors in general—have consequences for physical and mental health (Adebahr 2020). Furthermore, there is some evidence that the structure and quality of social ties predict morbidity and mortality, and that negative relationships are a source of conflicts (Brooks and Dunkel-Schetter 2011). High levels of social conflicts are associated with a dysregulation of the endocrine, cardiovascular, and the immune system and are, therefore, associated with a higher risk of negative health outcomes (Brooks and Dunkel-Schetter 2011). Since relationships do not change that often, older people who are exposed to forms of negative social ties seem to be particularly vulnerable to this special potential stressor, because—if applicable—they have been mostly exposed to it for a long time. Moreover, older adults could be particularly sensitive, because in older age the quality of social relationships could play a greater role for well-being due to changes in the structure and function of social ties (Brooks and Dunkel-Schetter 2011).

Although many attempts have been made to conceptualize and operationalize social support, the literature on negative forms of support is much narrower and different terms are used in this regard (Laireiter et al. 2007). Laireiter et al. (2007) use terms like social undermining, social negativity, as well as stressful, negative, or inadequate support. Brooks and Dunkel-Schetter 2011 point out that in addition to differences in terminology, the terms that are used, such as social undermining, negative social interactions, or problematic social ties refer to a similar phenomenon they summarize under the multi-dimensional construct social negativity. In our study, we used the term social burden according to Fydrich et al. (1999), who define social burden as the rather negative aspects of social support, which is associated with feelings of rejection, narrowing, criticism, and overwhelming hindering, as well as with negative aspects of social interactions. It is important to note that it has been suggested that negative aspects of social experiences could have a greater impact on health than the positive ones (Rook 1984). This so-called burden-oriented approach (in contrast to the resource-oriented approach) has come more and more in the focus of attention in recent years, although it is known that positive social relationships are essential for the satisfaction and independence of older adults and that social burden is a risk factor for healthy aging (Schneider 1995). However, although it seems plausible that social burden is a potential stressor in older age, research is rare, in which the associations between social burden as a type of negative social support and psychological stress and diurnal cortisol secretion have been investigated. Furthermore, it still remains an open question how social burden is related with cognitive aging.

**Fig. 1:**
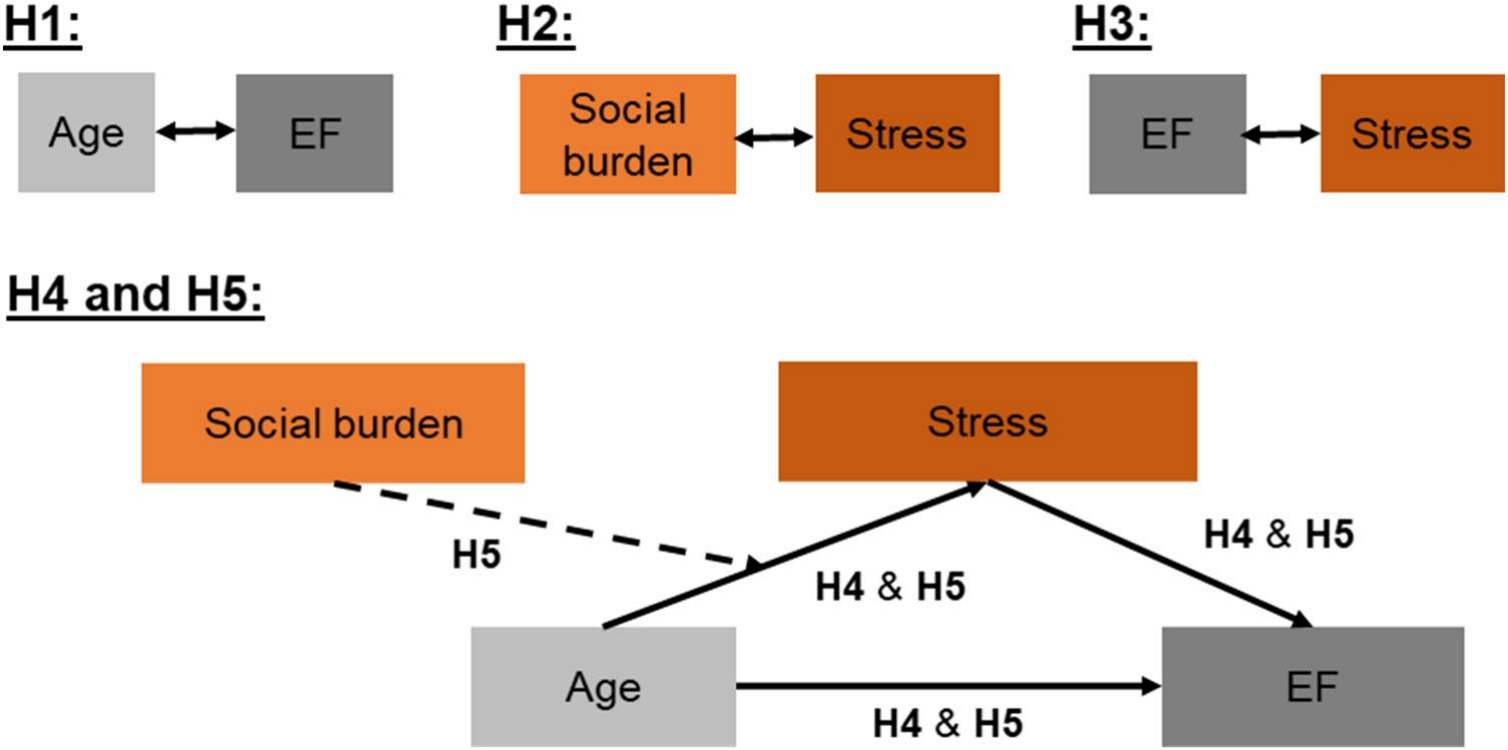
Visualization of the hypotheses (H1, H2, H3, H4, H5) that were tested in this study. As executive functions (EF), inhibition that was assessed by means of a Stroop task, and cognitive flexibility that was assessed by means of the Trail Making Test were considered. Perceived stress and two indices of the diurnal cortisol profiles (the area under the curve with respect to ground and the diurnal slope) were considered as stress measures

The aim of our study was to investigate the associations between social burden as a potential stressor and EF in older age. We referred to the two EF domains inhibition and cognitive flexibility, which are both important for an active and independent lifestyle in older age (Kelly et al. 2017; Wirth, Haase et al. 2014). We differentiated between perceived stress and diurnal cortisol profiles as a physiological measure of diurnal cortisol secretion patterns. Our first hypothesis (H1) was that older adults will show lower cognitive flexibility and inhibition. Because of our cross-sectional design, we referred to this association as a measure for cognitive aging. Our second hypothesis (H2) was that higher perceived levels of social burden will be associated with higher levels of perceived stress as well as with diurnal cortisol profiles. Third, we hypothesized (H3) that higher stress levels—perceived stress and cortisol measures—will be associated with lower EF (i.e., cognitive flexibility and inhibition). Our fourth hypothesis (H4) was that perceived stress and diurnal cortisol profiles are mediators for the association between age and cognitive flexibility as well as inhibition as markers for EF (i.e., cognitive aging). Furthermore, we assumed that social burden will be an additional stressor, which we—as our fifth hypothesis (H5)—added to the model underlying H4. Therefore, H5 was that social burden moderates the association between age and stress and, thus, indirectly cognitive aging. An overview about all hypotheses is provided in Fig. 1.

## Methods

### Participants

In this study, initially *N* = 100 older adults aged 64 years or older participated. Seventeen participants had to be excluded from statistical analysis, because four did not provide enough saliva for cortisol assessment, four reported taking glucocorticoid medication, eight because of outliers in the diurnal cortisol profiles (more than 3 standard deviations from the participants’ mean) and one because of missing data in the social burden questionnaire. The final sample consisted of *N* = 83 participants. Most of the participants were members in a senior leisure program of the ‘*Bavarian Red Cross*’ (Erlangen, Germany). All participants gave their written and informed consent. Data were anonymized directly after collection to protect participant’s privacy. The study was conducted according to the principles expressed in the Declaration of Helsinki and was approved by the local ethics committee of the Friedrich-Alexander University Erlangen-Nürnberg (# 194_18 B).

### Cognitive assessment

#### Dementia screening

The Mini Mental State Examination (MMSE; Folstein et al. 1975) was used for dementia screening. The MMSE assesses the cognitive domains orientation, memory, attention, arithmetic, memory, and language. It allows a classification into four categories: ‘no dementia’ (27–30), ‘mild dementia’ (20–26), ‘moderate dementia’ (10–19), and ‘severe dementia’ (≤ 9; Eschweiler et al. 2010; Folstein et al. 1975).

#### Inhibition

**Fig. 2:**
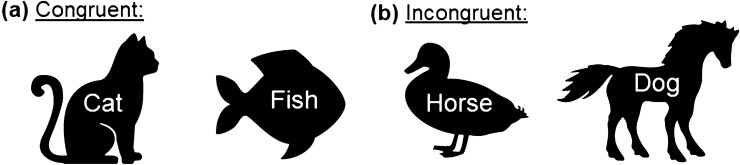
Example stimuli of the Stroop test: **a** congruent and **b** incongruent condition. In the original task, the German words ‘Fisch’ (fish), ‘Pferd’ (horse), ‘Hund’ (dog), ‘Ente’ (duck), and ‘Katze’ (cat) were used

For assessing inhibition, an in-house developed animal Stroop task was used (Stroop 1935; Fig. 2). Our animal Stroop task followed the same approach for inducing interference as the original color-word Stroop task (in which colors and words are used; Stroop 1935). With our task, we aimed to induce interference between animal shapes and overlayed animal words in the incongruent condition, in which animal shapes and words did not match (e.g., ‘fish’ printed on a dog). It can also be used for participants with colorblindness, which makes it more widely applicable. In our animal Stroop task, a word stimulus and an animal stimulus were presented simultaneously to the participants. The illustration of the animal was black, and the word was written in white letters and overlaid the animal’s body (Fig. 2). The participant’s task was to name the animal figures while ignoring (inhibiting) the overlaid animal names. Five animals were used in our study (fish, horse, dog, duck, and cat). The German names of these animals were used as overlays (Fisch = fish, Pferd = horse, Hund = dog, Ente = duck, and Katze = cat). Two conditions were used in our study, a congruent and an incongruent one. Prior to the actual testing, a practice sheet was presented to the participants, which included five animals each. Each testing sheet included five rows with four illustrations each, resulting in 20 animals per sheet. Each participant was given four sheets of each condition. Time was measured separately for each of the eight test sheets, using a stopwatch. The time measurement started immediately after handing out the sheet to the participant. The difference in the processing time between the incongruent and congruent condition was used as performance measure (Stroop score = time [Stroop incongruent condition]—time [Stroop congruent condition]). Larger interference scores were considered as indicators for poorer inhibition performance.

#### Cognitive flexibility

The Trail-Making Test (TMT; Reitan 1992), which is one of the most widely used international methods for brain functioning screening, was used to assess cognitive flexibility (Rodewald et al. 2012; Tischler and Petermann 2010). The TMT consists of the subtests A and B. Each one includes a sample task. Part A examines visuo-motor processing and visual processing speed. Part B assesses cognitive flexibility. The task is to connect 25 circles in the correct order, using the dominant hand. The circles include numbers (Part A) and numbers or letters (Part B). Depending on the level of neuropsychological functioning, the duration of the TMT is five to ten minutes (Tischler and Petermann 2010). The time measurement started immediately after the oral instruction of the experimenter. Time was measured by means of a stopwatch. If a mistake was made, the experimenter pointed this out and the participant had to correct it immediately (Rodewald et al. 2012; Tischler and Petermann 2010). The difference scores in processing time between both parts (TMT [B-A] = processing time [TMT-B]—processing time [TMT-A]) were used as performance measure for cognitive flexibility in our study (Arbuthnott and Frank 2000; Giovagnoli et al. 1996). Larger difference scores TMT [B-A] were considered as lower levels of cognitive flexibility in our study.

### Questionnaires

#### Depression

The Geriatric Depression Scale (GDS; Sheikh and Yesavage 1986) was used as a depression screening tool. It consists of 15 items in a yes/no-response format. The GDS allows a classification into three categories: ‘*no*’ (1–5), ‘*mild to moderate*’ (6–10), and ‘*severe depression*’ (11–15) (Sheikh and Yesavage 1986; Yesavage et al. 1982).

#### Perceived stress

The German 10-item version of the Perceived Stress Scale (PSS; Cohen et al. 1983; Klein et al. 2016) was used as a measure of perceived stress within the last month. The items are rated on a five-point scale from which a sum score is calculated with higher scores indicating higher levels of perceived stress.

#### Social burden

Social burden was assessed by means of the subscale ‘Social Burden’ from the ‘Questionnaire of Social Support’ [*Fragebogen zur Sozialen Unterstützung*] (Fydrich et al. 2007). This scale contains twelve items and measures the extent to which the person feels criticized, rejected, narrowed down, and overwhelmed by others (Hessel et al. 1998). The answer format is a five-point Likert scale (Fydrich et al. 2007). A mean value is calculated as measure for social burden with higher values indicating higher levels of perceived social burden.

### Saliva sampling and analysis

Saliva samples were collected by means of salivettes (Sarstedt, Nümbrecht, Germany). Participants were instructed to keep the salivette in their mouth for at least one minute and to move it back and forth, but not to chew on it. Participants were provided with eight salivettes and were instructed to collect four on each of two consecutive days within seven days after the cognitive assessment. The following time points were scheduled: immediately after awakening, before lunch, in the afternoon/before dinner, and at bedtime. Participants were instructed not to eat, drink, or brush their teeth at least 30 min before saliva collection saliva samples were stored in the participants fridge or freezer for approximately 1–2 weeks until they were given back to the experimenters and stored at -30 °C. For analysis, salivettes were thawed at room temperature and were centrifuged at 2000 g and 20 °C for ten minutes immediately before analysis. Salivary cortisol concentrations were determined in duplicate using enzyme-linked immunosorbent assays (ELISA, IBL, Hamburg, Germany). Intra- and inter-assay coefficients of variation were below 10%. The total diurnal cortisol output, which was calculated as area under the curve with respect to ground (AUCg; Pruessner, Kirschbaum, Meinlschmid, and Hellhammer 2003) as well as the diurnal slope, was used as markers for diurnal cortisol profiles. The AUCg can be used as a measure of total diurnal cortisol output, and the diurnal slope can be used as a marker for diurnal cortisol changes. Mean values from both days were calculated and used for further statistical analysis.

### General procedure

Participants were recruited by means of e-mails, flyers, and posters including information about the study. Furthermore, our study was advertised personally in a local senior leisure program. The aim of this program called “*Seniorennetz*,” which is offered by the ‘*Bavarian Red Cross*’ (Erlangen, Germany), is to provide older people access to the "digital world". The members attend age-appropriate courses, which are led by volunteer tutors. Additionally, meetings in groups of interests and one-to-one discussions about anything related to computers, tablets, and smartphones are offered. We recruited most of our participants this way. However, because of time restrictions, we were not able to only focus on this target group and also included other people who take not part in this program.

Our study was carried out between May and August 2019. At the day of cognitive testing, the participant and one experimenter (AP or SK) met in a quiet room, usually in the laboratory of the Chair for Health Psychology in Erlangen between 10 a.m. and 5 p.m. First, the declaration of consent was signed by the participants on that day. Subsequently, the experimenter gave instructions about the saliva collection procedure which the participants should do by themselves at their homes within the following week. After the introduction to saliva collection, the cognitive testing and the questionnaire assessment started. This included the Stroop test and the TMT (Reitan 1992) as well as the PSS (Cohen et al. 1983), the social burden subscale (Fydrich et al. 1999, 2007), the MMSE (Folstein et al. 1975), and the GDS (Yesavage et al. 1982). The order of cognitive testing was randomized between the participants. Finally, an appointment for the return of the saliva samples was scheduled. The whole session lasted between 90 min and two hours.

### Statistical data analysis

For statistical analyses, IBM SPSS Statistics (version 26) was used. Normality of distribution was tested by means of the Kolmogorov–Smirnov test. Because of positive skewness and violation of normality, cortisol-AUCg’s, difference score in the TMT and perceived social burden were transformed by means of the natural logarithm (ln) prior to further statistical analysis. Furthermore, PSS scores were transformed by means of the square root-transformation to achieve a normal distribution. Partial correlations were used for testing the first, second, and third hypotheses. Sex, BMI, and relationship status were included as covariates in the partial correlations as well as in all further statistical analyses. For the mediation and moderation analyses, the SPSS macro PROCESS (Hayes 2013; Hayes and Rockwood 2017) was used. For mediation analyses (hypothesis 4), the PROCESS model no. 4 was tested (*Y*: dependent variable, *X*: independent variable, *M*: mediator variable). The mediation analysis includes three steps: first, evaluating the direct path (i.e., investigating whether there is a total effect between *X* and *Y* without a mediator), second, evaluating the indirect paths (between *X* and *M* and between *M* and *Y*, respectively), and third, investigating whether the direct effect disappears when including the indirect path (*X*–*M*–*Y*) into the model. For the moderated mediation analysis (hypothesis 5), the PROCESS model no. 7 was tested (*Y*: dependent variable, *X*: independent variable, *W*: moderator variable, *M*: mediator variable). 10,000 bootstrap samples were used for both models. The data set that has been used for statistical analysis is provided as supplementary material S1. To correct for multiple comparisons, a Bonferroni-adjusted α-level of α_adjusted_ = 0.05/7 = 0.0071 was used for analyses, because seven variables (age, TMT performance, Stroop performance, PSS scores, social burden, cortisol-AUCg, and cortisol slope) were compared (Sinclair et al. 2013). However, because making decisions based on (non-) significance only has been increasingly criticized and because it has become more and more common to interpret effect sizes instead of *p*-values (e.g., Hubbard and Lindsay 2008), we still report results which fulfill the criterion *p*_uncorrected_ ≤ 0.05, but interpret them with caution. We considered correlation coefficients *r* between 0.1 and 0.2 as small effects, between 0.2 and 0.3 as medium effects, and > 0.3 as large effects (Gignac and Szodorai 2016).

## Results

### Sample characteristics

The final sample consisted of *N* = 83 participants (38 male, mean age = 74.0 ± 5.7 years, body-mass-index (BMI) = 25.5 ± 4.1 kg/m^2^). All participants were retired and in most cases without any part-time job. The mean score in the MMSE (Folstein et al. 1975) was 28.5 ± 1.2, and therefore, participants were classified as cognitively healthy. Furthermore, the GDS scores (Sheikh and Yesavage 1986) did not show clinically relevant values (1.4 ± 1.7). The intake of the following medication was reported: *N* = 29 thyroid medication, *N* = 18 beta-blocker, and *N* = 3 hormonal drugs. None of the variables that were investigated in this study (age, Stroop performance, TMT performance, PSS scores, social burden, cortisol-AUCg, and cortisol slope) differed between participants who took any of these medications and participants who did not report medication intake (all *p* ≥ 0.332). Therefore, medication was not included as covariate into the further statistical analyses.

**Fig. 3:**
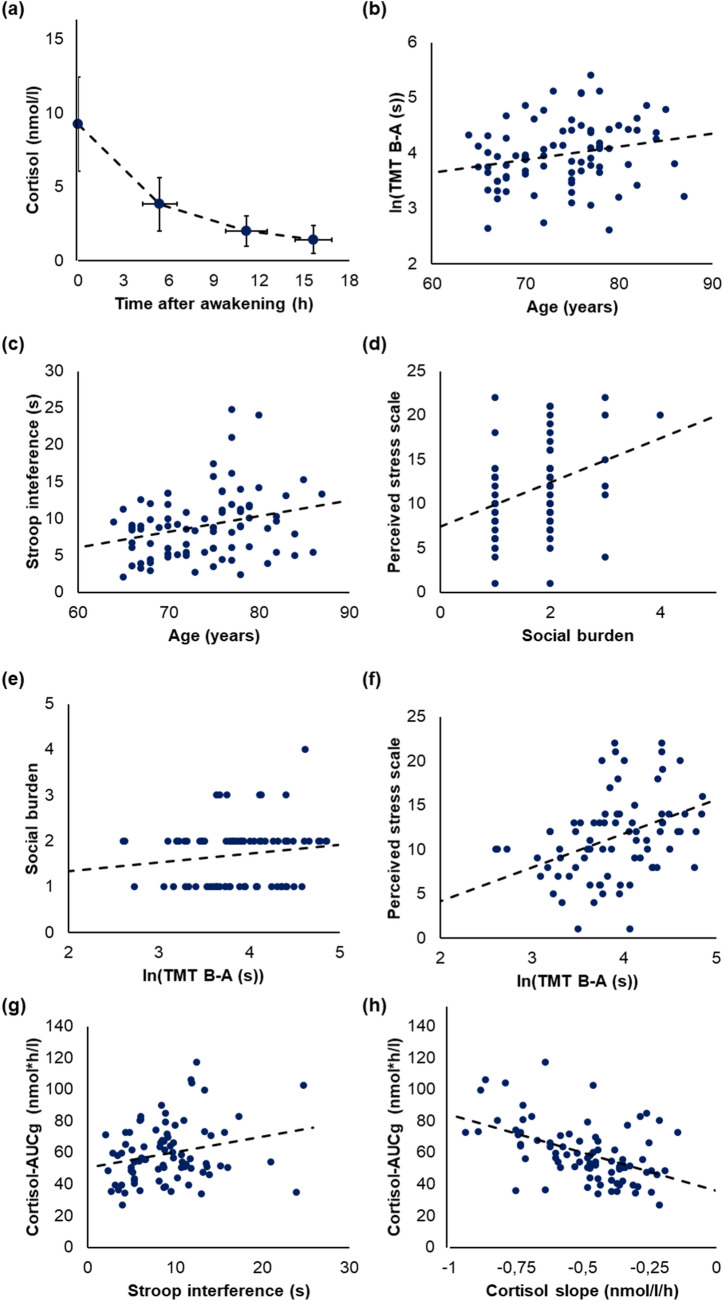
**a** Mean diurnal cortisol profiles, which were averaged over two consecutive days, **b**–**h** (marginally) significant correlations between age, cognitive performance in the Stroop task (as measure for inhibition) or Trail-Making Test (TMT; as measure for cognitive flexibility), perceived stress (PSS), social burden, and cortisol-area under the curve with respect to ground (AUCg). Note that transformed values of the AUCg, PSS, and social burden scale were used for statistical analysis

Mean cortisol profiles of all participants are shown in Fig. 3a and are summarized in Table 1. Mean cortisol-AUCgs were 59.7 ± 18.4 nmol*h/l, and mean diurnal slopes were −0.5 ± 0.2 nmol/l/h (both averaged over two consecutive days). The individual wake-up time (i.e., the time point of the first sample) ranged between 5:30 am and 9:00 a.m. and the individual bedtime (i.e., time point of the last sample) between 9:00 p.m. and midnight. Mean processing times in the TMT as measure for cognitive flexibility were 42.2 ± 16.5 s in Part A and 105.3 ± 51.2 s in Part B. Mean difference scores in the TMT were 63.1 ± 39.6 s. The latter are higher than mean values that have been reported in a validation study by Rodewald et al. 2012, who reported mean processing times of 40.2 ± 11.9 s for Part A and 76.0 ± 23.6 s for Part B. However, the participants in our study were up to 87 years old and the age limit in the study by Rodewald et al. 2012 was 84 years.

**Table 1:** Mean cortisol levels (averaged over two consecutive days), performance in the Stroop task as measure for inhibition, performance in the Trail-Making Test (TMT) as measure for cognitive flexibility, as well as perceived stress and social burden scores

	Mean	Standard deviation
Cortisol t_0_ (nmol/l)	9.2	3.2
Cortisol t_1_ (nmol/l)	3.8	1.8
Cortisol t_2_ (nmol/l)	2.0	1.0
Cortisol t_3_(nmol/l)	1.4	0.9
Cortisol-AUCg (nmol*h/l)	59.7	18.4
Cortisol slope (nmol/l/h)	−0.5	0.2
Stroop congruent (s)	13.4	3.2
Stroop incongruent (s)	22.4	5.9
Stroop interference (s)	9.1	4.5
TMT-A (s)	42.2	16.5
TMT-B (s)	105.3	51.2
TMT-B–TMT-A (s)	63.1	39.6
Perceived stress scale	11.7	5.2
Social burden score	1.7	0.6

Mean processing times in the Stroop task as measure for inhibition were 13.4 ± 3.2 s in the congruent and 22.4 ± 5.9 s in the incongruent condition. Mean Stroop interference scores were 9.1 ± 5.1 s. Because an in-house-developed Stroop task was used, no reference data are available. Mean Stroop interference scores were significantly higher in men than in women (*t*_(56.6)_ = -2.35; *p* = 0.023; women: 8.0 ± 3.2 s; men: 10.4 ± 5.5). However, this association did not remain significant after adjusting the α-level for multiple comparisons (α_adjusted_ = 0.0071). No further sex differences were found for any of the other variables (all *p* ≥ 0.121).

Overall, participants showed relatively low levels of perceived stress (11.7 ± 5.2) and social burden (1.7 ± 0.6). Fifty-nine (71.1%) of the participants reported having an intimate partnership and the others (*N* = 24, 28.9%) reported being single. Most (*N* = 56, 67.5%) were living together with the partner or their family, *N* = 26 (31.3%) were living alone, and one (1.2%) was living in a rest home.

### ***Correlational analyses (hypotheses 1***–***3)***

Partial correlations in which sex, BMI, and relationship status were included as covariates, yielded the following results: age was positively correlated with the Stroop and TMT scores, indicating that older participants showed lower performance in the Stroop task as measure for inhibition as well as in the TMT as measure for cognitive flexibility (Stroop: *r*_(78)_ = 0.24, *p* = 0.038; TMT: *r*_(78)_ = 0.23, *p* = 0.043; Fig. 3b and c), which is in line with our first hypothesis (H1). However, this association did not remain significant after adjusting the α-level for multiple comparisons (α_adjusted_ = 0.0071).

Our second hypothesis (H2) was confirmed: social burden was positively related with perceived stress (*r*_(78)_ = 0.38, *p* < 0.001; Fig. 3d). However, this was found for the cortisol indices (AUCg and cortisol slope; *p* ≥ 0.732).

Our third hypothesis (H3) that participants who perceived more stress or show higher cortisol-AUCgs and flatter cortisol slopes will show lower cognitive performance in the domains cognitive flexibility (which was assessed by means of the TMT) and inhibition (which was assessed by means of a Stroop task) was confirmed for the association between TMT and perceived stress (PSS and TMT: *r*_(78)_ = 0.44, *p* < 0.001; Fig. 3e). No associations were found for the Stroop task and, thus, not for inhibition (*p* ≥ 0.351). Furthermore, no associations between any of the cortisol indices and any of the EF domains (cognitive flexibility or inhibition) were found.

Further correlation analyses, for which we had no specific hypotheses, showed that social burden was positively associated with performance in the TMT (*r*_(78)_ = 0.24, *p* = 0.031; Fig. 3f). However, this association did not remain significant when using the adjusted α-level α_adjusted_ = 0.0071. Moreover, both cortisol indices (AUCg and cortisol slope) and, thus, both measures for diurnal cortisol secretion were associated with age: Older participants showed lower cortisol-AUCgs (*r*_(78)_ = 0.38, *p* < 0.001; Fig. 3h) and diurnal cortisol slopes were negatively associated with cortisol-AUCgs (*r*_(78)_ = -0.49, *p* < 0.001).

The whole correlation matrix is provided in Table 2.

**Table 2:** Partial correlations (controlled for sex, body-mass index, and relationship status) between age, Stoop performance as measure for inhibition, performance in the Trail-Making Test as measure for cognitive flexibility, perceived stress, and the two cortisol indices. Significant correlations (*p* < .05) are highlighted in bold

		**1**	**2**	**3**	**4**	**5**	**6**	**7**
1. Age	*r*	1	**.23°**	**.23°**	.15	.01	−.30**	.19
	*p*		**.038**	**.043**	.180	.904	**.007**	.100
2. Stroop	*r*		1	.17	.07	−.12	.20	.04
	*p*			.124	.564	.351	.080	.745
3. TMT	*r*			1	.44***	**.24°**	.05	.05
	*p*				** < .001**	**.031**	.637	.639
4. PSS	*r*				1	.38***	.07	.11
	*p*					** < .001**	.565	.330
5. Social burden	*r*					1	.02	−.04
	*p*						.892	.732
6. Cortisol-AUCg	*r*						1	−.49***
	*p*							** < .001**
7. Cortisol slope	*r*							1
	*p*							

*°p* < .05 (not adjusted α-level)

*TMT* Trail-Making Test, *PSS* Perceived Stress Scale, *AUCg* area under the curve with respect to ground

**p < .007 (adjusted α-level), ***p < .001

### Stress as a mediator of cognitive aging with respect to the domains inhibition and cognitive flexibility (hypothesis 4)

To test our fourth hypothesis that stress (perceived stress or cortisol measures) mediates the association between age and EF, separate mediation analyses were calculated, for both EF domains inhibition and cognitive flexibility.

#### Inhibition

**Fig. 4:**
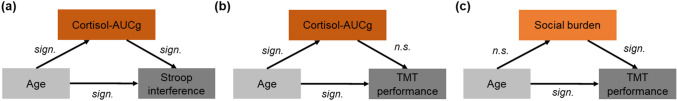
Significant (sign.) and non-significant (n.s.) paths in the mediation models for testing hypothesis 4

For inhibition, which was assessed by means of a Stroop task, the mediation model was confirmed when including the cortisol-AUCg as mediator variable (Fig. 4a and Table 3): The direct path between age and Stroop performance (*p* = 0.038), the paths between age and cortisol-AUCg (*p* = 0.0073), as well as between the cortisol-AUCg and Stroop performance (*p* = 0.010), were significant. However, this association did not pass the correction for multiple comparisons (α_adjusted_ = 0.0071). Furthermore, the whole indirect pathway (age—cortisol-AUCg—Stroop) was significant (CI [-0.15, -0.01]). This means that the association between age and Stroop performance (i.e., lower performance in older age) is associated with higher cortisol levels in the older participants. However, when including the diurnal cortisol slope or perceived stress into the model, no relevant significant associations were found.

**Table 3:** Regression coefficients for the mediation model that tested the associations between age as independent variable, performance in the Stroop task (as measure for inhibition) as dependent variable, and the cortisol-AUCg as mediator (H4). Significant associations (*p* < .05) are highlighted in bold

	*R*^2^	*B*/Effect	(*M*)*SE*	*F/t*	*p*	CI
*Step 1*						
ln(cortisol-AUCg)	0.10		0.09	2.05	.095	
**(Constant)**		4.91	0.45	10.84	<.001***	**[4.01, 5.82]**
**Age**		−0.02	0.01	−2.76	.007**	**[−0.03, −0.005]**
BMI		0.01	0.01	0.88	.380	[−0.01, 0.23]
Sex		0.08	0.07	1.08	.283	[−0.07, 0.23]
Relationship status		0.04	0.08	0.44	.659	[−0.12, 0.20]
*Step 2*						
**Stroop interference**	0.21		17.29	4.06	.003**	
**(Constant)**		−29.95	10.11	−2.96	.004**	**[−50.09, −9.81]**
**Age**		0.25	0.09	2.88	.005**	**[0.08, 0.43]**
ln(cortisol-AUCg)		4.20	1.60	2.63	**.010°**	**[1.02, 7.38]**
BMI		0.09	0.11	0.78	.436	[−0.14, 0.32]
Sex		1.47	1.05	1.40	.165	[−0.61, 3.55]
Relationship status		−0.83	1.14	−0.72	.469	[−3.09, 1.44]
*Step 3:*						
**Direct effect**		0.18	0.09	2.11	**.038°**	**[0.01, 0.36]**
**Direct effect (indirect paths included)**		0.25	0.09	2.88	.005**	**[0.07, 0.43]**
**Indirect effect**		−0.07	0.04			**[−0.2, −0.005]**^**sign**^

*°p* < .05 (not adjusted α-level)

*sign*. Significant, *AUCg* area under the curve with respect to ground, *ln* natural logarithm, *BMI* body-mass-index

**p < .007 (adjusted α-level), ***p < .001

#### Cognitive flexibility

For cognitive flexibility, which was assessed by means of the TMT, the following significant associations were found for the cortisol-AUCg (Fig. 4b and Table 4): The direct path between age and TMT performance (*p* = 0.043) and the indirect path between age and the cortisol-AUCg reached significance (*p* = 0.007). Note that the association between age and TMT performance did not pass the correction for multiple comparisons. The path between the cortisol-AUCg and the TMT-performance as well as the whole indirect path (age—cortisol-AUCg—TMT) were not significant (CI [-0.01, 0.003]). Therefore, the hypothesis that the diurnal cortisol output is a mediator for cognitive aging (i.e., cognitive flexibility, assessed by means of the TMT) was not confirmed. For the diurnal cortisol slope, no relevant significant associations were found as well.

**Table 4:** Regression coefficients for the mediation model that tested the associations between age as independent variable, performance in the TMT (as measure for cognitive flexibility) as dependent variable, and the cortisol-AUCg as mediator (H4. Significant associations (*p* < .05) are highlighted in bold )

	*R*^2^	*B*/Effect	(*M*)*SE*	*F/t*	*p*	CI
*Step 1*						
ln(cortisol-AUCg)	0.10		0.09	2.50	.095	
**(Constant)**		4.91	0.45	10.84	< .001***	**[4.01, 5.82]**
**Age**		−0.02	0.01	−2.76	.007**	**[−0.03, −0.005]**
BMI		0.01	0.01	0.88	.380	[−0.01, 0.02]
Sex		0.80	0.07	1.08	.283	[−0.07, 0.23]
Relationship status		0.36	0.08	0.44	.659	[−0.12. 0.20]
*Step 2*						
TMT score	0.11		0.32	1.93	.099	
(Constant)		1.06	1.38	0.76	.447	[−1.70, 3.82]
**Age**		0.03	0.01	2.31	**.024°**	**[0.004, 0.05**]
ln(cortisol-AUCg)		0.25	0.22	1.15	.252	[−0.18, 0.69]
BMI		0.00	0.02	0.20	.844	[−0.03, 0.03]
Sex		0.05	0.14	0.35	.725	[−0.23, 0.34]
Relationship status		−0.24	0.16	−1.53	.130	[−0.55. 0.07]
*Step 3*						
**Direct effect**		0.03	0.01	2.05	**.044°**	**[0.001, 0.047]**
**Direct effect (indirect paths included)**		0.03	0.01	2.31	**.024°**	**[0.004, 0.022]**
Indirect effect		0.00	0.00			[−0.01. 0.003]

*°p* < .05 (not adjusted α-level)

*TMT* Trail-Making Test, *AUCg* area under the curve with respect to ground, *ln* natural logarithm, *BMI* body-mass index

**p < .007 (adjusted α-level), ***p < .001

### Social burden as a moderator of the mediation of stress on cognitive aging with respect to the domains inhibition and cognitive flexibility (hypothesis 5).

The aim of our fifth hypothesis was to test whether social burden is an additional stressor that moderates the association between age, EF, and stress that has been investigated in H4. However, because of the small sample size the following findings should be treated with caution and should be considered as preliminary.

**Fig. 5:**
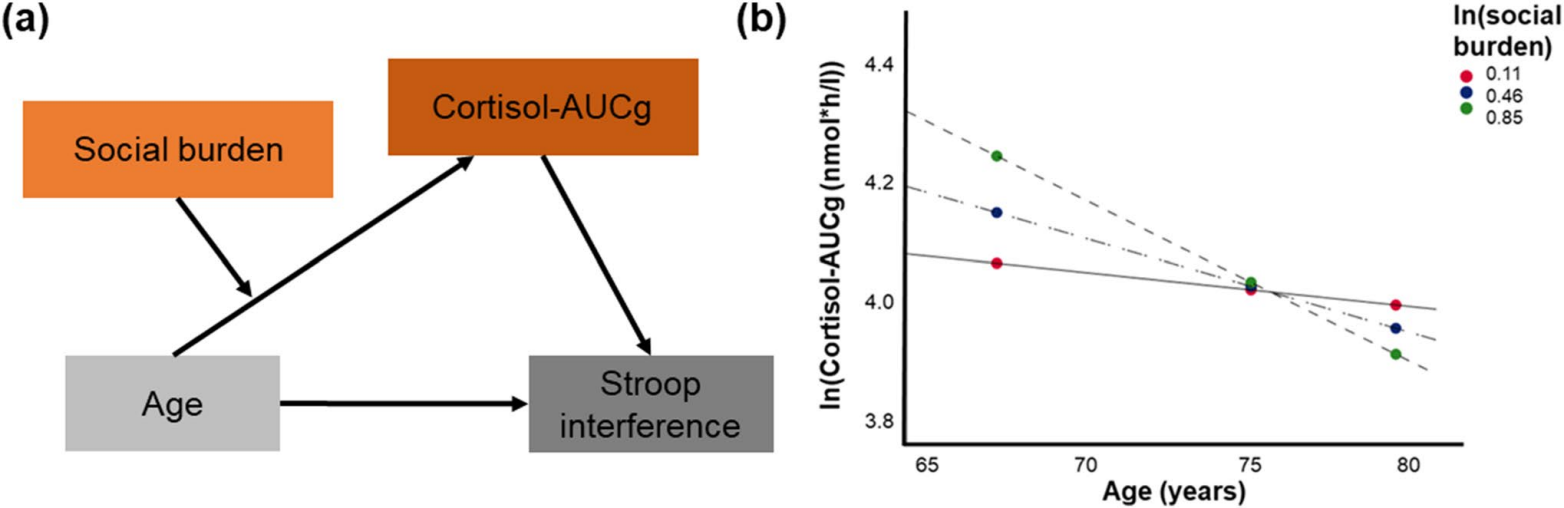
**a** Moderated mediation model between age, Stroop performance, cortisol-AUCg, and social burden, and **b** three-way interaction age*cortisol-AUCg*social burden

Because the mediation models H4 were significant for the cortisol-AUCg only, the model was only tested for this variable and not for perceived stress. For inhibition, the following significant associations were found (Fig. 5a, Table 5): Stroop interference as measure for inhibition was associated with the cortisol-AUCg (*p* = 0.01) as well as with age even when the indirect path was included in the model (*p* = 0.005) as well as. The cortisol-AUCg was only marginally significantly associated with social burden (*p* = 0.099). However, this *p*-value is much higher than our adjusted α-level (α_adjusted_ = 0.0071). The indirect path (age—cortisol-AUCg—Stroop) was significant for participants with high and medium levels of social burden only (CI [−0.22, −0.01] for ln(social burden = 0.85 and CI [−0.14, 0.0.003] for ln(social burden) = 0.46), but not for participants with low levels of social burden (CI [−0.11, 0.06] for ln(social burden = 0.11; Fig. 5b). This three-way interaction reflects that social burden was particularly relevant in the younger participants and that only high and medium levels of perceived social burden interacted with age and the cortisol-AUCg. When using the same model, no relevant significant associations were found for the TMT scores as measure for cognitive flexibility.

**Table 5:** Regression coefficients for the moderated mediation model that tested the associations between age as independent variable, cognitive flexibility as dependent variable, the cortisol-AUCg as mediator, and social burden as moderator (H5). Significant associations (*p* < .05) are highlighted in bold

	*R*^2^	*B*/Effect	(*M*)*SE*	*F/t*	*p*	CI
*Step 1*						
ln(cortisol-AUCg)	0.13		0.09	1.85	.101	
**(Constant)**		3.95	0.73	5.40	< .001***	**[2.50, 5.41]**
Age		0.002	0.01	−0.22	.825	[−0.02, 0.02]
ln(social burden)°		2.11	1.26	1.67	.099	[−0.41, 4.6]
Age*ln(social burden)		−0.03	0.02	−1.66	.101	[−0.06, 0.01]
BMI		0.01	0.01	0.67	.505	[−0.01, 0.02]
Sex		0.05	0.08	0.65	.518	[−0.10, 0.20]
Relationship status		0.02	0.08	0.26	.797	[−0.14, 0.18]
*Step 2*						
**Stroop interference**	0.21		17.29	4.06	.003**	
**(Constant)**		−29.95	10.11	−2.96	.004**	**[−50.09, −9.81]**
**Age**		0.25	0.09	2.88	.005**	**[0.08, 0.43]**
**ln(cortisol-AUCg)**		4.20	1.60	2.63	**.010°**	**[1.02, 7.38]**
BMI		0.09	0.11	0.78	.436	[−0.14, 0.31]
Sex		1.47	1.05	1.40	.165	[−0.61, 3.55]
Relationship status		−0.83	1.14	−0.73	.469	[−3.09, 1.44]
*Step 3*						
**Direct effect**		0.25	0.09	2.88	.005**	[0.08, 0.43]
**Indirect effect**	**ln(social burden)**					
	0.11	−0.02	0.04			[−0,11, 0.06]
	**0.46**	−0.06	0.04			**[−0,14, −0.003]**^**sign**^
	**0.85**	−0.11	0.05			**[−0,22, −0,01]**^**sign**^

*°p* < .05 (not adjusted α-level)

*sign*. Significant, *AUCg* area under the curve with respect to ground, *ln* natural logarithm, *BMI* body-mass index

**p < .007 (adjusted α-level), ***p < .001

## Discussion

In this study, we investigated the role of social burden as a potential additional stressor in older age and its associations with cognitive aging (i.e., with associations between age and cognitive flexibility and inhibition as parts of EF). Perceived stress was considered as psychological stress measure, and diurnal cortisol profiles were considered. Our main findings were that social burden was highly correlated with perceived stress and that the total diurnal cortisol output was a mediator for the relationship between age and inhibition (which was assessed by means of a Stroop task). Furthermore, social burden interacted with the association between age and the cortisol-AUCg in the latter model, in the way that especially in younger older age, high and medium levels of social burden are associated with higher cortisol levels and, thus, with lower cognitive performance.

Older participants showed lower EF (i.e., lower cognitive flexibility and inhibition) with medium effect sizes (*r* > 0.2), and thus, our first hypothesis H1 was confirmed. However, this association did not pass the correction for multiple comparisons. Nevertheless, the direction is in line with our hypothesis and a large number of previous studies (e.g., MacPherson et al. 2002) and (in principle, although not statistically significant after correction for multiple comparisons) contradicts the not so widespread assumption that EF—in contrast with memory—does not or only in some specific tests decrease in older age (e.g., Crawford et al. 2000).

Furthermore, we were able to show that social burden was highly correlated (*p* < 0.001 and strong effect size *r* > 0.3) with perceived stress in general, highlighting its importance as a stressor which has been reported previously (Cranford 2004) and supporting our second hypothesis H2. However, social burden was not directly associated with any of the diurnal cortisol indices in our study. Moreover, social burden was directly associated with cognitive flexibility, which was assessed by means of the TMT, indicating that participants who reported more perceived social burden showed lower cognitive flexibility. However, this association did not pass the correction for multiple comparisons but had a medium effect size (*r* > 0.2).

Moreover, we found that participants who reported higher levels of perceived stress showed lower performance in the TMT (as measure for cognitive flexibility), which is in favor of our hypothesis H4. This finding fits well into previous research that also found lower cognitive performances for older adults with higher levels of perceived stress (e.g., Aggarwal et al. 2014; Chen, Wang et al. 2018; Korten et al. 2017; Turner et al. 2017). However, this was not found for the Stroop task as measure for inhibition in our study.

Furthermore, we found that the total diurnal cortisol output was a mediator for the association between age and inhibition, confirming the in our hypothesis H4 postulated association between cortisol levels and cognition in older age. This is in line with previous findings from several cross-sectional and longitudinal studies in which higher HPA-axis activity was associated with poorer cognitive performance in healthy as well as in cognitively impaired older adults (e.g., Arsenault-Lapierre et al. 2010; Evans et al. 2011; Li et al. 2006). Furthermore, links between higher subjective stress and poorer cognitive performance have been reported previously (e.g., Johansson et al. 2010; Liston et al. 2009; Wild-Wall et al. 2009). At last, we found that the mediating role of cortisol interacts with social burden in the way that especially in younger older age, high levels of social burden are associated with higher cortisol levels and, therefore, with lower cognitive performance, which is in favor of our H5. However, it should not be left unmentioned that other directions of the associations between age, stress, and cognition are conceivable. For example, with respect to the human capital hypothesis (Mirowsky and Ross 1998) and findings on associations between cognitive processes and coping flexibility (Cheng and Cheung 2005), an alternative explanation might that people with better cognitive flexibility are more capable to available resources and adaptive coping strategies under stress.

An important finding of our study is that the physiological measures were associated with inhibition, but not with cognitive flexibility, and vice versa with respect to subjective stress (i.e., that an association with cognitive flexibility, but not with inhibition was found for perceived stress). Differences between subjective stress measures and physiological stress markers have been reported previously (Goldman et al. 2005; Oldehinkel et al. 2011), but the underlying mechanisms are not yet fully understood. Our findings highlight the importance of including both subjective and physiological measures in research on the associations between stress and cognitive aging.

Overall, we were able to show the importance of considering social burden as an additional stressor, which fosters age-related stress processes that are associated with cognitive decline. This can be contrasted well with the approaches that can be found in resource-oriented support research, in which, for example, social support (e.g., by friends and family members) is considered as a resource or as a protective factor (Kessler, Price, & Wortman 1985; Lepore et al. 1991). The burden-oriented approach that our study relies on, has been coming more and more in the research focus in recent years. Associations between higher levels of negative aspects of social ties and basal cortisol levels and other physiological markers and mortality have been found in previous studies in older and breast cancer samples (Friedman et al. 2012; Kroenke et al. 2012; Seeman et al. 2002). Furthermore, it has been suggested that negative aspects of social experiences can have a greater impact on health than the positive ones (Rook 1984). In contrast, other authors have shown that the number of supportive relationships was a stronger predictor of psychological distress than the number of negative ones (Uchino et al. 2004). Laireiter et al. 2007 point out that “*The extent to which positive expressions [of certain]… variables… are able to buffer negative support can be assumed on the basis of the results of stress research (Perrez* et al. 2005*), but has not been empirically researched any more than the question of whether this can also be provided by positive support*” [*Inwieweit positive Ausprägungen [bestimmter] … Variablen … in der Lage sind, Negativunterstützung abzupuffern, kann auf Grund der Ergebnisse der Stressforschung zwar angenommen werden (Perrez* et al. 2005*), ist aber empirisch genauso wenig erforscht, wie die Frage, ob dies auch positive Unterstützung vermag*] (Laireiter et al. 2007, p. 47)]. Therefore, both the negative and the positive aspects of social relationships and their interactions should be considered in future research and the negative aspects should not be neglected.

Our study is subject to some further limitations which are especially related to our definition of social burden as the negative aspects of social support as well as the corresponding feelings of rejection, narrowing, criticism, and overwhelming obstacles, as well as the negative aspects of social interactions (Fydrich et al. 1999, 2007). Some other attempts to conceptualize and operationalize social burden have been suggested (e.g., Brooks and Dunkel-Schetter, 2011; Laireiter et al. 2007). For example, Laireiter et al. 2007 describe social burden as social undermining, social negativity, and stressful, negative or inadequate support. However, Brooks & Dunkel-Schetter, 2011point out that in addition to differences in terminology, terms such as social undermining, negative social interactions, or problematic social ties refer to a similar phenomenon that can be summarized as social negativity as a multi-dimensional construct. Therefore, all these mentioned further aspects of social negativity should be considered in future research. Another limitation of our study is that because of the cross-sectional design, no causal conclusions can be drawn. Furthermore, the representativity of the sample appears to be limited because all participants were cognitively healthy, had high educational levels, and showed no signs of dementia or other forms of cognitive decline. Furthermore, most of our participants were integrated in regular social activities and most of them participated in a German leisure program and were in an intimate relationship. Especially the participation in the regional leisure time program makes it difficult to adequately consider important confounders. Both factors (the leisure time program and the social relationships) might have been a social resource and might be the reason why only low to medium levels of social burden and perceived stress were reported by our participants. However, even in this sample we found that social burden is associated with perceived stress and indirectly with cognitive aging, which highlights the importance of our findings. Nevertheless, other samples with higher levels of social burden and participants who are not engaged in social activities and who do not in most cases live in an intimate partnership should be investigated in future research. Moreover, the number of participants was 83 only, and therefore, statistical power for the complex model in hypothesis H5 was low. Therefore, the (non-) findings from testing H5 should be treated with caution and should be considered as preliminary findings, which must be verified in future research with larger sample sizes. Furthermore, the small sample size might be the reason that some of the associations that were found and that had at least a medium effect size did not remain significant after correcting for multiple comparisons. Nevertheless, with respect to the extensive methods and great effort, the number is relatively large in relation to comparable studies.

Furthermore, we used a stopwatch for measurement of the processing times during the cognitive testing. Although it has to be kept in mind that this adds the individual reaction time of the experimenter to the measured time, it has been shown that the intrasession reliability is high for stopwatch usage (Bohannon 1995). Because we used difference scores (between part B and part A of the TMT and between the incongruent and the congruent condition of the Stroop task), it is unlikely that our results with respect to the cognitive performance measures were strongly influenced by the time measurement procedure.

In particular, with respect to practical implications of our study, due to the demographic change, as well as the associated supply and refinancing problems, focusing on our research question seems to be important (Esch 2002; World Health Organization 2015). The findings of our study, which highlight the associations between stress and cognition in older age, as well as previous findings related to stress-associated health outcomes (e.g., Sapolsky 2004), suggest the necessity to develop stress-reducing interventions for this age group. As one alternative, relaxation trainings or strengthening of health behaviors have been suggested (e.g., Reif et al. 2018; Renner and Staudinger 2008). Moreover, EF should be directly targeted, e.g., through cognitive trainings. Beside a gain for those affected, a possible side effect of introducing such interventions specifically targeted to older populations could be a long-term cost reduction for the health-care system (Esch 2002).

In future research, our study should be replicated in a larger, non-regional sample that is exposed to a higher number of or higher intensities of social burden and that is less engaged in social activities and social relationships. Furthermore, no individual attitudes, motives, or potentially protective factors that are associated with positive social relationships as mediators of the stress measures were included in this work, which, however, have been considered as important factors in previous studies (Blaney 2013; Reimann and Pohl 2006; Sapolsky 2004). The inclusion of the personality traits and temperament, which also appear to be significant resources, should be considered in future research. Furthermore, longitudinal designs should be used to investigate the time course of the findings and whether they are permanent or temporary. Moreover, participants with cognitive impairments should be considered in future research.

## Conclusions

To summarize, we were able to show that social burden is a highly relevant additional stressor in older age. Furthermore, we found that social burden interacts with the diurnal cortisol output, which mediates cognitive aging. Our findings highlight the importance of considering social burden in the study of cognitive functioning in older adults. We conclude that both practitioners and researchers in the field of cognitive aging should also focus on the negative aspects of social relationships (in the sense of a burden-oriented approach) and not only on the positive ones.

## Supplementary Information

Below is the link to the electronic supplementary material.

S1. Data set that has been used for statistical analysis. (CSV 8 kb)

## References

[CR1] Adam EK, Quinn ME, Tavernier R, McQuillan MT, Dahlke KA, Gilbert KE (2017). Diurnal cortisol slopes and mental and physical health outcomes: a systematic review and meta-analysis. Psychoneuroendocrinology.

[CR2] Adebahr P (2020). Negative Beziehungsaspekte und gesundheitliche Ungleichheiten. In: *Soziale Netzwerke und gesundheitliche Ungleichheiten* , pp 87–107. Springer

[CR3] Aggarwal NT, Wilson RS, Beck TL, Rajan KB, de Leon CFM, Evans DA, Everson-Rose SA (2014). Perceived stress and change in cognitive function among adults aged 65 and older. Psychosom Med.

[CR4] Arbuthnott K, Frank J (2000). Trail making test, part B as a measure of executive control: validation using a set-switching paradigm. J Clin Exp Neuropsychol.

[CR5] Arnsten AFT (2009). Stress signalling pathways that impair prefrontal cortex structure and function. Nat Rev Neurosci.

[CR6] Arsenault-Lapierre G, Chertkow H, Lupien S (2010). Seasonal effects on cortisol secretion in normal aging, mild cognitive impairment and Alzheimer’s disease. Neurobiol Aging.

[CR7] Belanoff JK, Gross K, Yager A, Schatzberg AF (2001). Corticosteroids and cognition. J Psychiatr Res.

[CR8] Blaney PH (2013). Stress and depression ln adults: a critical review. Stress Coping.

[CR9] Bohannon RW (1995). Stopwatch for measuring thumb-movement time. Percept Mot Skills.

[CR10] Brand M, Markowitsch HJ (2004). Frontalhirn und Gedächtnis im Alter NeuroGeriatrie.

[CR11] Brooks KP, Dunkel-Schetter C (2011). Social negativity and health: conceptual and measurement issues. Soc Pers Psychol Compass.

[CR12] Chen Y, Wang J, Liang Y, Sun F, Dong X (2018). Perceived stress and cognitive functions among Chinese older adults: the moderating role of health status. Gerontol Geriat Med.

[CR13] Cheng C, Cheung MWL (2005). Cognitive processes underlying coping flexibility: differentiation and integration. J Pers.

[CR14] Chrousos GP (2009). Stress and disorders of the stress system. Nat Rev Endocrinol.

[CR15] Chrousos GP, Gold PW (1998). A healthy body in a healthy mind—and vice versa—the damaging power of “uncontrollable” stress. J Clin Endocrinol Metab.

[CR16] Cohen S, Kamarck T, Mermelstein R (1983). A global measure of perceived stress. J Health Social Behav.

[CR17] Cranford JA (2004). Stress-buffering or stress-exacerbation? Social support and social undermining as moderators of the relationship between perceived stress and depressive symptoms among married people. Pers Relat.

[CR18] Crawford JR, Bryan J, Luszcz MA, Obonsawin MC, Stewart L (2000). The executive decline hypothesis of cognitive aging: do executive deficits qualify as differential deficits and do they mediate age-related memory decline?. Aging Neuropsychol Cogn.

[CR19] DeCarli C, Massaro J, Harvey D, Hald J, Tullberg M, Au R, Wolf PA (2005). Measures of brain morphology and infarction in the framingham heart study: establishing what is normal. Neurobiol Aging.

[CR20] Esch T (2002). Health in stress: change in the stress concept and its significance for prevention, health and life style. Gesundheitswesen.

[CR21] Esch T, Stefano GB, Fricchione GL, Benson H (2002). The role of stress in neurodegenerative diseases and mental disorders. Neuro Endocrinol Lett.

[CR22] Eschweiler GW, Leyhe T, Klöppel S, Hüll M (2010). Neue Entwicklungen in der Demenzdiagnostik. Deutsches Ärzteblatt.

[CR23] Evans PD, Fredhoi C, Loveday C, Hucklebridge F, Aitchison E, Forte D, Clow A (2011). The diurnal cortisol cycle and cognitive performance in the healthy old. Int J Psychophysiol.

[CR24] Folstein MF, Folstein SE, McHugh PR (1975). Mini-mental state. A practical method for grading the cognitive state of patients for the clinician. J Psychiatr Res.

[CR25] Friedman EM, Karlamangla AS, Almeida DM, Seeman TE (2012). Social strain and cortisol regulation in midlife in the US. Soc Sci Med.

[CR26] Fydrich T, Geyer M, Hessel A, Sommer G, Brähler E (1999). Social support questionnaire (F-SozU): Norms of a representative sample. Diagnostica.

[CR27] Fydrich T, Sommer G, and Brähler E (2007) Fragebogen zur sozialen Unterstützung. Hogrefe, F-SozU

[CR28] Gaab J, Rohleder N, Nater UM, Ehlert U (2005). Psychological determinants of the cortisol stress response: the role of anticipatory cognitive appraisal. Psychoneuroendocrinology.

[CR29] Gignac GE, Szodorai ET (2016). Effect size guidelines for individual differences researchers. Personality Individ Differ.

[CR30] Giovagnoli AR, Del Pesce M, Mascheroni S, Simoncelli M, Laiacona M, Capitani E (1996). Trail making test: normative values from 287 normal adult controls. Italian J Neurol Sci.

[CR31] Goldman N, Glei DA, Seplaki C, Liu I-W, Weinstein M (2005). Perceived stress and physiological dysregulation in older adults. Stress.

[CR32] Gunnar MR, Vazquez DM (2001). Low cortisol and a flattening of expected daytime rhythm: Potential indices of risk in human development. Dev Psychopathol.

[CR33] Harada CN, Love MCN, Triebel KL (2013). Normal cognitive aging. Clin Geriatr Med.

[CR34] Hayes AF (2013) Mediation, moderation, and conditional process analysis. Introduction to mediation, moderation, and conditional process analysis: a regression-based approach Edn. Guilford Publications, New York, pp 1–20

[CR35] Hayes AF, Rockwood NJ (2017). Regression-based statistical mediation and moderation analysis in clinical research: observations, recommendations, and implementation. Behav Res Ther.

[CR36] Heaney JLJ, Phillips AC, Carroll D (2010). Ageing, depression, anxiety, social support and the diurnal rhythm and awakening response of salivary cortisol. Int J Psychophysiol.

[CR37] Hessel A, Geyer M, Brähler E (1998). Soziale Unterstützung im Alter-Normierung des Fragebogens zur sozialen Unterstützung (F-SOZU) bei über 60jährigen. Z Klin Psychol Psychopathol Psychother.

[CR38] Hubbard R, Lindsay RM (2008). Why P values are not a useful measure of evidence in statistical significance testing. Theory Psychol.

[CR39] Johansson L, Guo X, Waern M, Östling S, Gustafson D, Bengtsson C, Skoog I (2010). Midlife psychological stress and risk of dementia: a 35-year longitudinal population study. Brain.

[CR40] Juster R-P, McEwen BS, Lupien SJ (2010). Allostatic load biomarkers of chronic stress and impact on health and cognition. Neurosci Biobehav Rev.

[CR41] Kelly ME, Duff H, Kelly S, Power JEM, Brennan S, Lawlor BA, Loughrey DG (2017). The impact of social activities, social networks, social support and social relationships on the cognitive functioning of healthy older adults: a systematic review. Syst Control Found Appl.

[CR42] Kessler RC, Price RH, Wortman CB (1985). Social factors in psychopathology: Stress, social support, and coping processes. Annu Rev Psychol.

[CR43] Klein EM, Brähler E, Dreier M, Reinecke L, Müller KW, Schmutzer G, Beutel ME (2016). The German version of the Perceived Stress Scale–psychometric characteristics in a representative German community sample. BMC Psychiatry.

[CR44] Korten NCM, Comijs HC, Penninx BW, Deeg DJH (2017). Perceived stress and cognitive function in older adults: which aspect of perceived stress is important?. Int J Geriatr Psychiatry.

[CR45] Kroenke CH, Michael Y, Tindle H, Gage E, Chlebowski R, Garcia L, Caan BJ (2012). Social networks, social support and burden in relationships, and mortality after breast cancer diagnosis. Breast Cancer Res Treat.

[CR46] Laireiter A-R, Fuchs M, Pichler M-E (2007). Negative soziale Unterstützung bei der Bewältigung von Lebensbelastungen: Eine konzeptuelle und empirische Analyse. Zeitschrift Für Gesundheitspsychologie.

[CR47] Lazarus RS, Folkman S (1984). *Stress, appraisal, and coping*.

[CR48] Lazarus RS, Folkman S (1987). Transactional theory and research on emotions and coping. Eur J Pers.

[CR49] Lepore SJ, Evans GW, Schneider ML (1991). Dynamic role of social support in the link between chronic stress and psychological distress. J Pers Soc Psychol.

[CR50] Li G, Cherrier MM, Tsuang DW, Petrie EC, Colasurdo EA, Craft S, Wilkinson CW (2006). Salivary cortisol and memory function in human aging. Neurobiol Aging.

[CR51] Liston C, McEwen BS, Casey BJ (2009). Psychosocial stress reversibly disrupts prefrontal processing and attentional control. Proc Natl Acad Sci.

[CR52] Lupien SJ, McEwen BS, Gunnar MR, Heim C (2009). Effects of stress throughout the lifespan on the brain, behaviour and cognition. Nat Rev Neurosci.

[CR53] MacPherson SE, Phillips LH, Della Sala S (2002). Age, executive function and social decision making: a dorsolateral prefrontal theory of cognitive aging. Psychol Aging.

[CR54] McEwen BS (1998). Stress, adaptation, and disease: Allostasis and allostatic load. Ann N Y Acad Sci.

[CR55] McEwen BS, Nasca C, Gray JD (2016). Stress effects on neuronal structure: hippocampus, amygdala, and prefrontal cortex. Neuropsychopharmacology.

[CR56] Mika A, Mazur GJ, Hoffman AN, Talboom JS, Bimonte-Nelson HA, Sanabria F, Conrad CD (2012). Chronic stress impairs prefrontal cortex-dependent response inhibition and spatial working memory. Behav Neurosci.

[CR57] Miller DB, O’callaghan, J. P. (2005). Aging, stress and the hippocampus. Ageing Res Rev.

[CR58] Miller GE, Chen E, Zhou ES (2007). If it goes up, must it come down? Chronic stress and the hypothalamic-pituitary-adrenocortical axis in humans.

[CR59] Mirowsky J, Ross CE (1998). Education, personal control, lifestyle and health: A human capital hypothesis. Res Aging.

[CR60] Miyake A, Friedman NP (2012). The nature and organization of individual differences in executive functions: Four general conclusions. Curr Dir Psychol Sci.

[CR61] Murmu MS, Salomon S, Biala Y, Weinstock M, Braun K, Bock J (2006). Changes of spine density and dendritic complexity in the prefrontal cortex in offspring of mothers exposed to stress during pregnancy. Eur J Neurosci.

[CR62] Oldehinkel AJ, Ormel J, Bosch NM, Bouma EMC, van Roon AM, Rosmalen JGM, Riese H (2011). Stressed out? Associations between perceived and physiological stress responses in adolescents: the TRAILS study. Psychophysiology.

[CR63] Perrez M, Laireiter A, and Baumann U (2005) Stress und coping als Einflussfaktoren. In: Lehrbuch Klinische Psychologie-Psychotherapie (3., erw. Aufl.). Hans Huber, pp 272–304

[CR64] Petermann H, and Roth M (2006) Alter: Produktiver Umgang mit den Aufgaben einer Lebensphase. In: Gesundheitspsychologie. Springer, pp 245–263

[CR65] Pfefferbaum A, Rohlfing T, Rosenbloom MJ, Chu W, Colrain IM, Sullivan EV (2013). Variation in longitudinal trajectories of regional brain volumes of healthy men and women (ages 10 to 85 years) measured with atlas-based parcellation of MRI. Neuroimage.

[CR66] Pruessner JC, Hellhammer DH, Kirschbaum C (1999). Burnout, perceived stress, and cortisol responses to awakening. Psychosom Med.

[CR67] Pruessner JC, Kirschbaum C, Meinlschmid G, Hellhammer DH (2003). Two formulas for computation of the area under the curve represent measures of total hormone concentration versus time-dependent change. Psychoneuroendocrinology.

[CR68] Reif J, Spieß E, Stadler P (2018). Effektiver umgang mit stress.

[CR69] Reimann S and Pohl J (2006) Stressbewältigung. In: *Gesundheitspsychologie*. Springer, pp 217–227

[CR70] Reinhardt EG (2007) Endokrinologische Veränderungen (Cortisol und Amylase) im Speichel bei akutem und chronischem Stress während einer stationären Psychotherapie.

[CR71] Reitan RM (1992) Trail making test. Reitan Neuropsychol Lab

[CR72] Renner B and Staudinger UM (2008) Gesundheitsverhalten alter Menschen

[CR73] Rodewald K, Bartolovic M, Debelak R, Aschenbrenner S, Weisbrod M, and Roesch-Ely D (2012) Eine Normierungsstudie eines modifizierten Trail Making Tests im deutschsprachigen Raum. Zeitschrift Für Neuropsychologie

[CR74] Rook KS (1984). The negative side of social interaction: impact on psychological well-being. J Pers Soc Psychol.

[CR75] Sapolsky RM (2004) Why zebras don’t get ulcers: The acclaimed guide to stress, stress-related diseases, and coping-now revised and updated. Holt paperbacks

[CR76] Schneider HD (1995). Die soziale Umwelt im Alter als Ressource oder als Belastung?. In: Psychologie der Lebensalter. Springer, pp 263–269

[CR77] Seeman TE, Singer BH, Ryff CD, Love GD, Levy-Storms L (2002). Social relationships, gender, and allostatic load across two age cohorts. Psychosom Med.

[CR78] Sheikh JI, and Yesavage JA (1986) Geriatric Depression Scale (GDS): recent evidence and development of a shorter version. Clin Gerontol J Aging Mental Health.

[CR79] Sinclair J, Taylor PJ, Hobbs SJ (2013). Alpha level adjustments for multiple dependent variable analyses and their applicability—a review. Int J Sports Sci Eng.

[CR80] Stalder T, Kirschbaum C, Kudielka BM, Adam EK, Pruessner JC, Wüst S, Hellhammer DH (2016). Assessment of the cortisol awakening response: expert consensus guidelines. Psychoneuroendocrinology.

[CR81] Stroop JR (1935). Studies of interference in serial verbal reactions. J Exp Psychol.

[CR82] Tischler L, Petermann F (2010). Trail making test (TMT). Z Psychiatr Psychol Psychother.

[CR83] Turner AD, James BD, Capuano AW, Aggarwal NT, Barnes LL (2017). Perceived stress and cognitive decline in different cognitive domains in a cohort of older African Americans. Am J Geriatr Psychiatry.

[CR84] Uchino BN, Holt-Lunstad J, Smith TW, Bloor L (2004). Heterogeneity in social networks: A comparison of different models linking relationships to psychological outcomes. J Soc Clin Psychol.

[CR85] Veldhuis JD, Sharma A, Roelfsema F (2013). Age-dependent and gender-dependent regulation of hypothalamic-adrenocorticotropic-adrenal axis. Endocrinol Metab Clin.

[CR86] Wahl HW, Schilling O (2012). Das hohe Alter. Entwicklungspsychologie.

[CR87] Wild-Wall N, Gajewski P, Falkenstein M (2009). Cognitive competence of older workers. Z Gerontol Geriatr.

[CR88] Wirth M, Haase CM, Villeneuve S, Vogel J, Jagust WJ (2014). Neuroprotective pathways: lifestyle activity, brain pathology, and cognition in cognitively normal older adults. Neurobiol Aging.

[CR89] Trends in maternal mortality: 1990–2015: estimates from WHO. World Health Organization, UNICEF, UNFPA, World Bank Group and the United Nations Population Division

[CR90] Yesavage JA, Brink TL, Rose TL, Lum O, Huang V, Adey M, Leirer VO (1982). Development and validation of a geriatric depression screening scale: a preliminary report. J Psychiatr Res.

[CR91] Young EA, Abelson J, Lightman SL (2004). Cortisol pulsatility and its role in stress regulation and health. Front Neuroendocrinol.

[CR92] Zsoldos, E., & Ebmeier, K. P. (2016). Aging and psychological stress. In Stress: concepts, cognition, emotion, and behaviour. Elsevier, pp 311–323

